# Supplementary material for: Clinical Phenotypes of Dual Kidney Transplant Recipients in the United States as Identified through Machine Learning Consensus Clustering
Source: Medicina (Kaunas). 2022 Dec 12;58(12):1831. doi: 10.3390/medicina58121831 (PMC9783488; doi:10.3390/medicina58121831)
Supplement: Supplementary file 1 [file medicina-58-01831-s001.zip › medicina-2018020-supplementary.pdf]

eMethods

Cluster derivation

We applied an unsupervised ML approach to develop clinical phenotypes of DKT recipients in the UNOS/OPTN database by conducting unsupervised consensus clustering.[1] We performed consensus clustering analysis on the whole study population. We initially assessed the distribution and missingness in phenotyping variables. Subsequently, missing data were imputed through multiple imputation using multivariate imputation by chained equations (MICE),[2] and non-normal data were z-score normalized. Multiple imputation is a widely used approach to estimate variables when data are missing at random. MICE is optimal when less than 30% of a variable's data are missing.[3-8] All of the extracted variables in our study had missing data less than 5%. We subsequently applied clustering using the consensus cluster algorithm. The algorithm begins by subsampling a proportion of items and a proportion of features from a data matrix. Each subsample is then partitioned into up to groups (k) by a user-specified clustering algorithm. This process is repeated for a specified number of times. Pairwise consensus values, defined as 'the proportion of clustering runs in which two items are grouped together, are calculated and stored in a consensus matrix (CM) for each cluster. Clustering settings used were as follows: maximum number of clusters, 10; number of iterations, 100; subsampling fraction, 0.8; clustering algorithm, , K-means; Euclidean distance).[1] The number of potential clusters ranges from 2 to 10, to avoid producing an excessive number of clusters that would not be clinical useful. Pairwise consensus values, defined as 'the proportion of clustering runs in which two items are [grouped] together[1], are calculated and stored in a CM for each k. Then for each k, a final agglomerative hierarchical consensus clustering using distance of 1−consensus values is completed and pruned to k groups, which are called consensus clusters.

The clustering algorithm is to maximize the potential number of clusters while maintaining high cluster consensus. The optimal number of clusters was determined by examining the CM heat map, cumulative distribution function, and cluster-consensus plots with the within-cluster consensus scores.[9, 10] The within-cluster consensus score, ranging between 0 and 1, is defined as the average consensus value for all pairs of individuals belonging to the same cluster.[10] A value closer to one indicates better cluster stability.[10] To examine the cluster profile, we calculated and graphically displayed the standardized mean differences of the variables between each cluster and the overall study population. Calculation of the standardized difference of each parameter used the cutoff of ±0.3 to show subgroup features with the key features for each cluster [11-22].

All cluster derivation analyses were performed using R, version 4.0.3 (RStudio, Inc., Boston, MA; http://www.rstudio.com/), with the packages of ConsensusClusterPlus (version 1.46.0)[10]. We imputed missing data through multivariable imputation by chained equation (MICE) method.[2] All analyses were two-tailed, and P value < .05 was considered statistically significant.

Figure S1. Consensus matrix heat map (k = 2) depicting consensus values on a white to blue color scale of each cluster

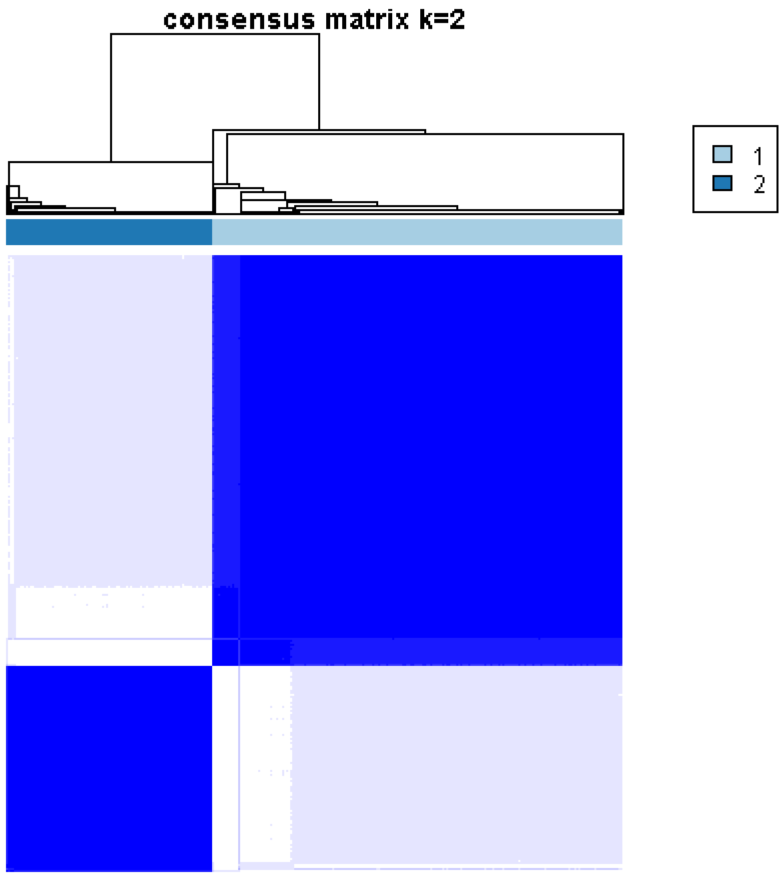

Figure S2. Consensus matrix heat map (k = 3) depicting consensus values on a white to blue color scale of each cluster

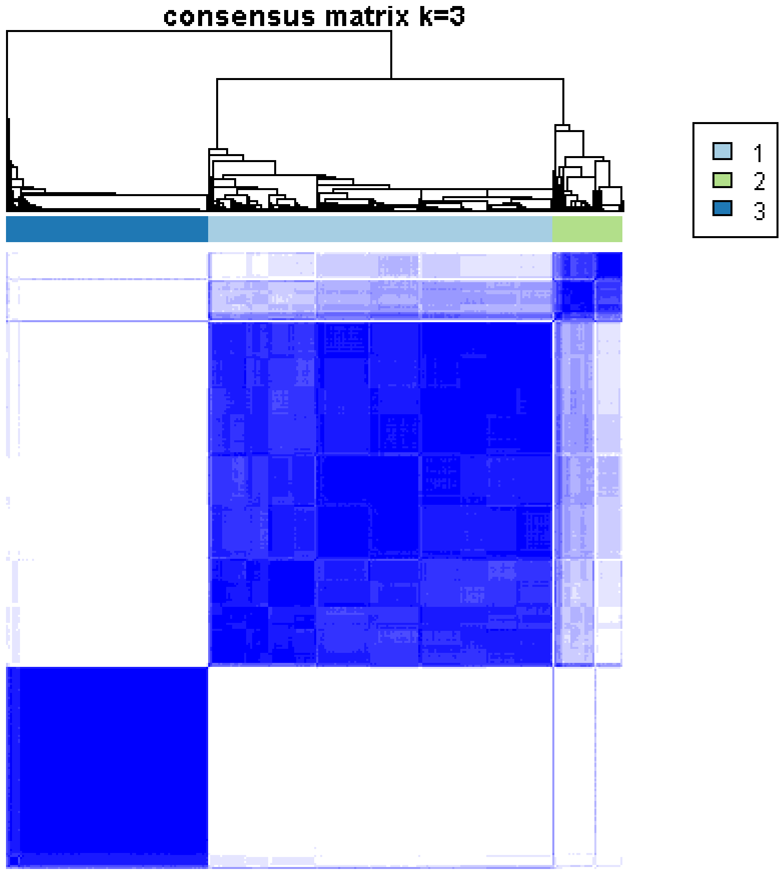

Figure S3. Consensus matrix heat map (k = 4) depicting consensus values on a white to blue color scale of each cluster

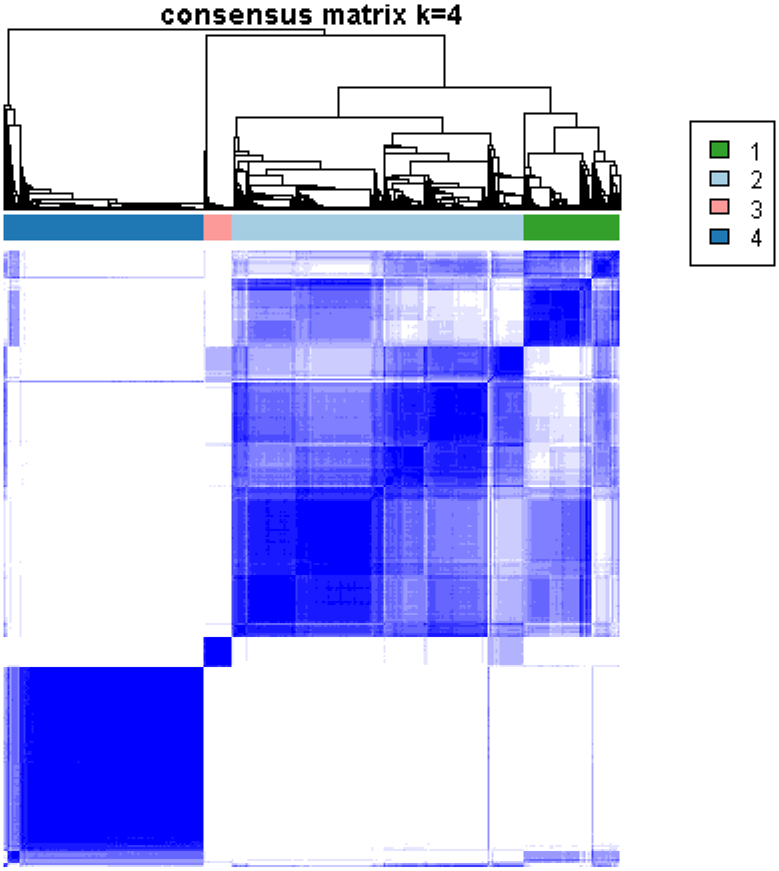

Figure S4. Consensus matrix heat map (k = 5) depicting consensus values on a white to blue color scale of each cluster

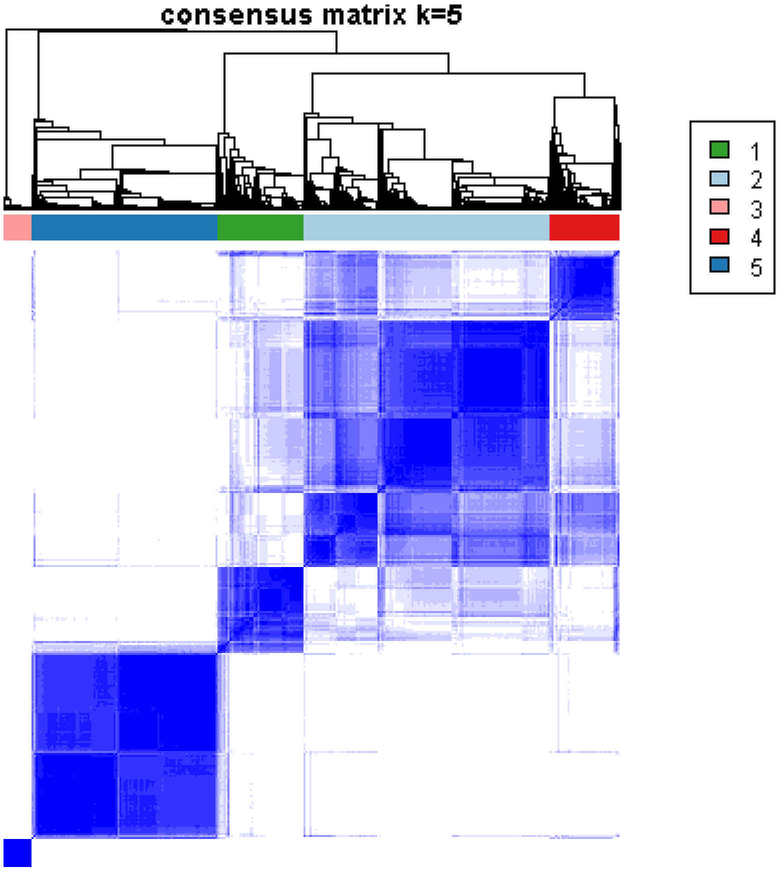

Figure S5. Consensus matrix heat map (k = 6) depicting consensus values on a white to blue color scale of each cluster

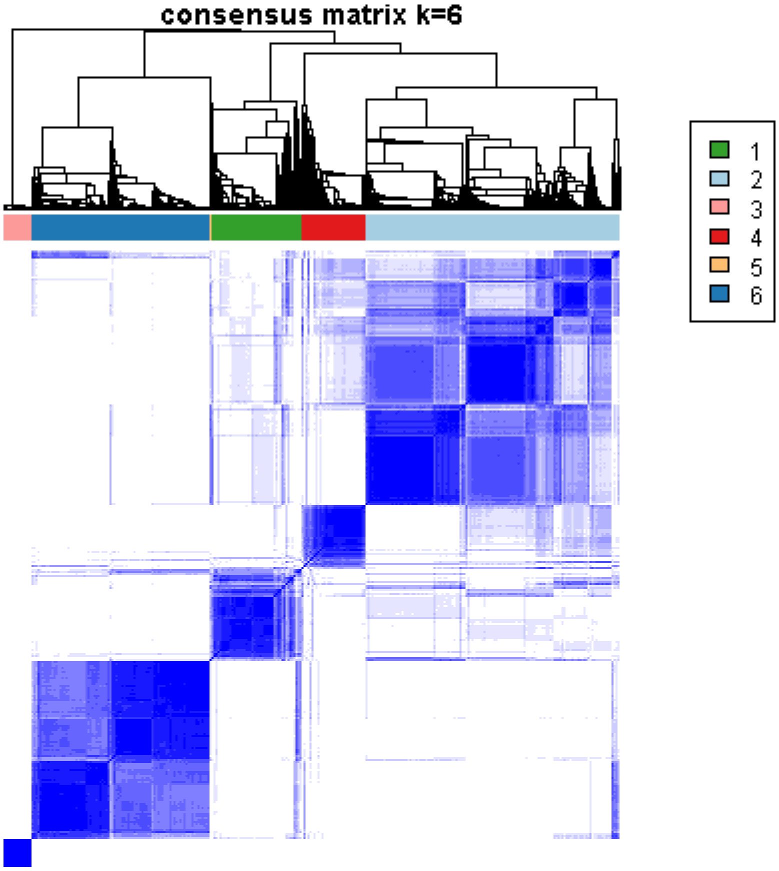

Figure S6. Consensus matrix heat map (k = 7) depicting consensus values on a white to blue color scale of each cluster

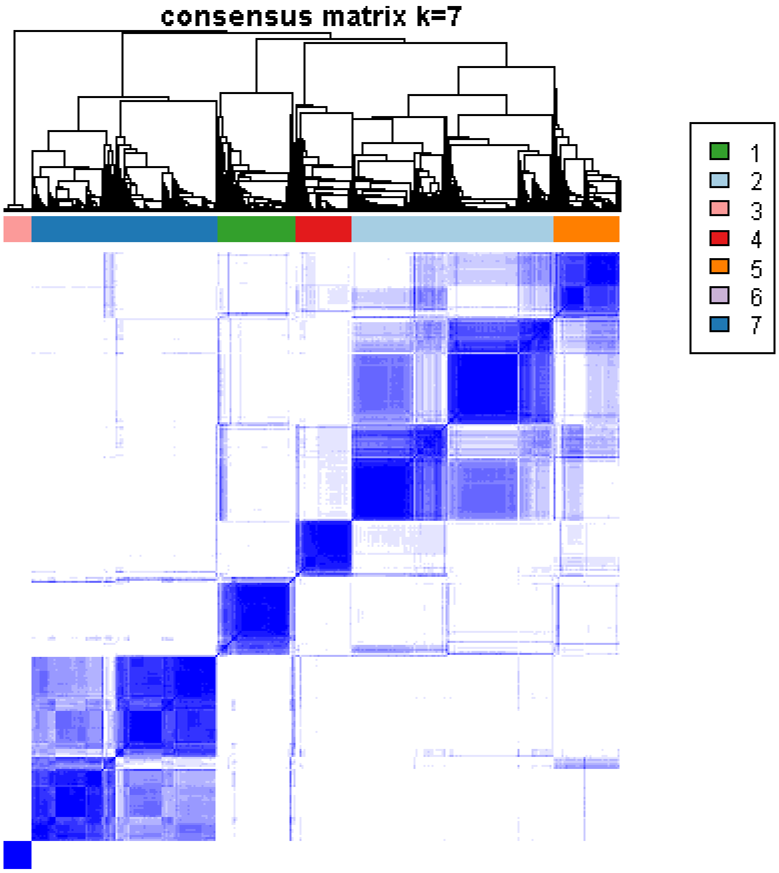

Figure S7. Consensus matrix heat map (k = 8) depicting consensus values on a white to blue color scale of each cluster

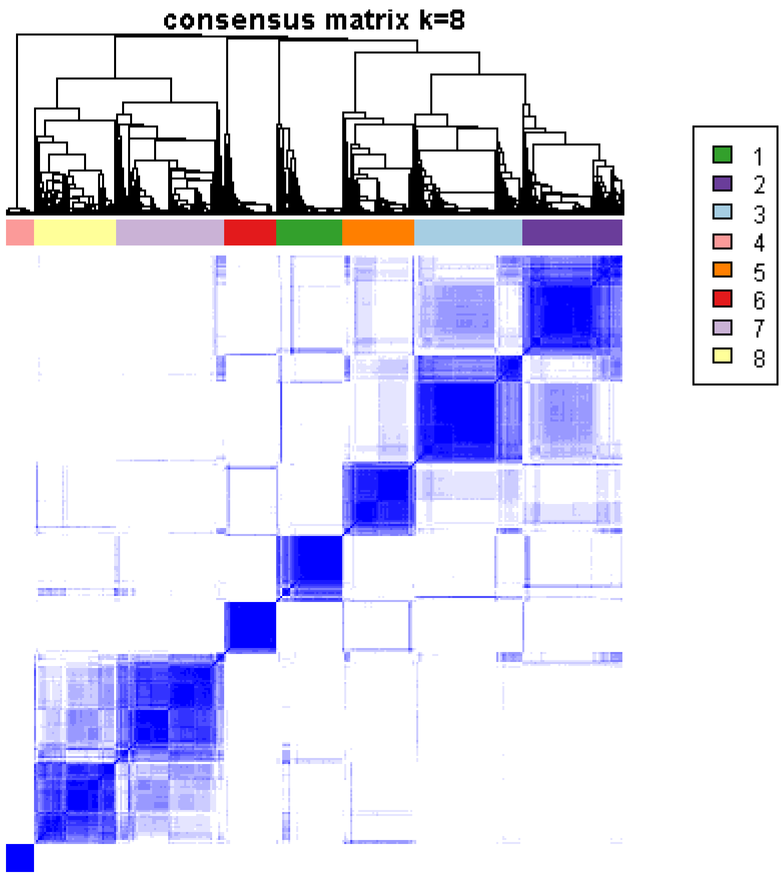

Figure S8. Consensus matrix heat map (k = 9) depicting consensus values on a white to blue color scale of each cluster

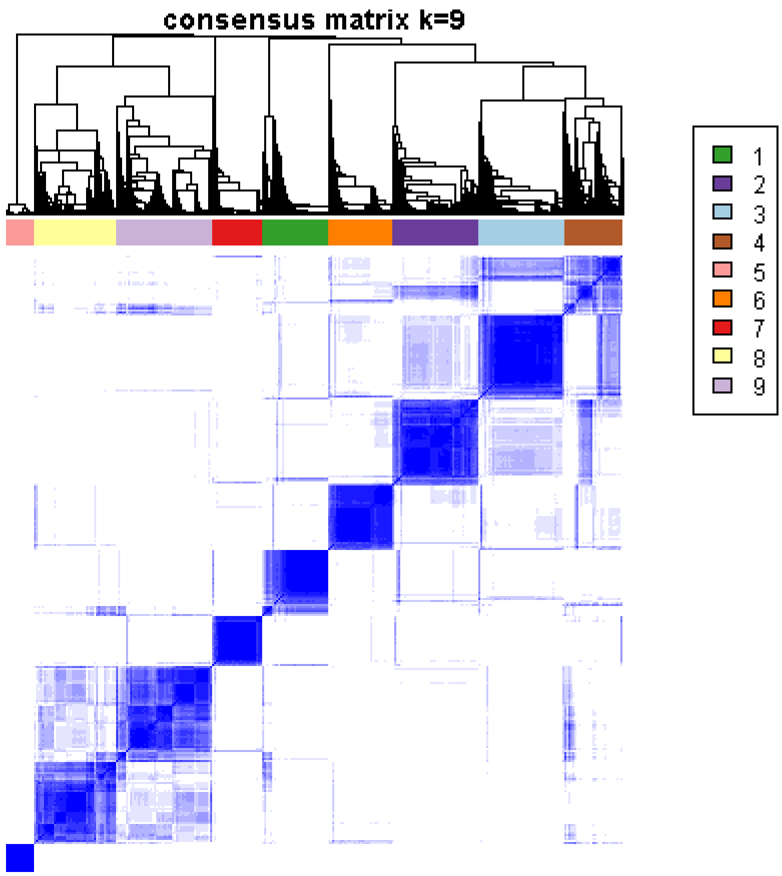

Figure S9. Consensus matrix heat map (k = 10) depicting consensus values on a white to blue color scale of each cluster

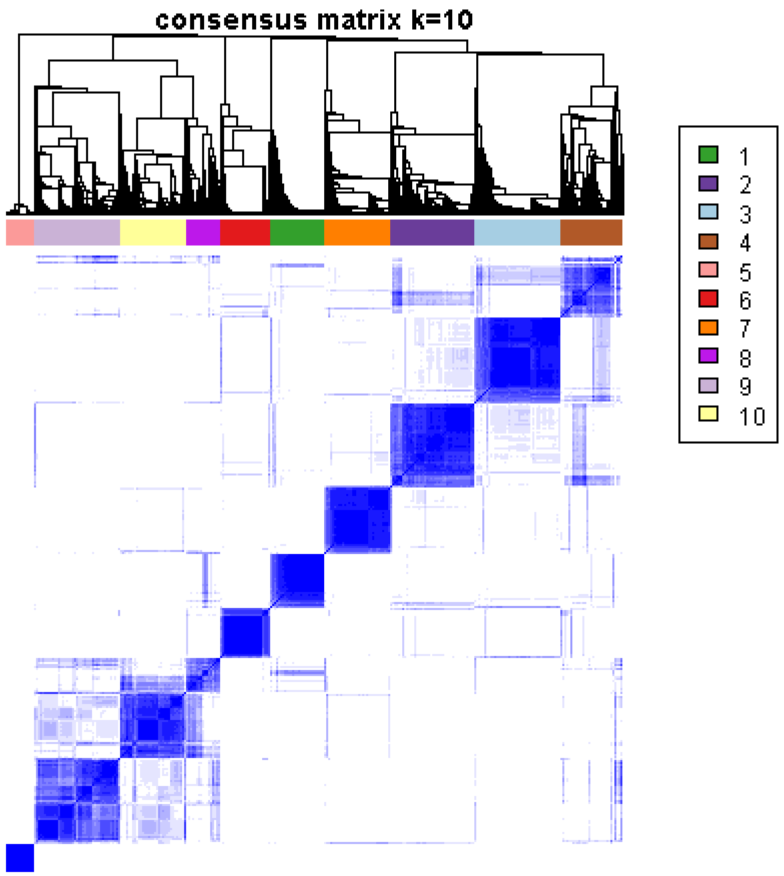

References

1 Monti S, Tamayo P, Mesirov J, Golub T. Consensus clustering: a resampling-based method for class discovery and visualization of gene expression microarray data. Machine learning. 2003: 91
2 Van Buuren S, Groothuis-Oudshoorn K. mice: Multivariate imputation by chained equations in R. Journal of statistical software. 2011: 1
3 Jannat-Khah DP, Unterbrink M, McNairy M, Pierre S, Fitzgerald DW, Pape J, Evans A. Treating loss-to-follow-up as a missing data problem: a case study using a longitudinal cohort of HIV-infected patients in Haiti. BMC Public Health. 2018: 1269 [PMID: 30453995 10.1186/s12889-018-6115-0: 10.1186/s12889-018-6115-0]
4 Knol MJ, Janssen KJ, Donders AR, Egberts AC, Heerdink ER, Grobbee DE, Moons KG, Geerlings MI. Unpredictable bias when using the missing indicator method or complete case analysis for missing confounder values: an empirical example. J Clin Epidemiol. 2010: 728 [PMID: 20346625 10.1016/j.jclinepi.2009.08.028: 10.1016/j.jclinepi.2009.08.028]
5 White IR, Carlin JB. Bias and efficiency of multiple imputation compared with complete-case analysis for missing covariate values. Stat Med. 2010: 2920 [PMID: 20842622 10.1002/sim.3944: 10.1002/sim.3944]
6 White IR, Royston P, Wood AM. Multiple imputation using chained equations: Issues and guidance for practice. Stat Med. 2011: 377 [PMID: 21225900 10.1002/sim.4067: 10.1002/sim.4067]
7 Hedden SL, Woolson RF, Carter RE, Palesch Y, Upadhyaya HP, Malcolm RJ. The impact of loss to follow-up on hypothesis tests of the treatment effect for several statistical methods in substance abuse clinical trials. J Subst Abuse Treat. 2009: 54 [PMID: 19008067 10.1016/j.jsat.2008.09.011: 10.1016/j.jsat.2008.09.011]
8 Donders ART, Van Der Heijden GJ, Stijnen T, Moons KG. A gentle introduction to imputation of missing values. Journal of clinical epidemiology. 2006: 1087
9 Șenbabaoğlu Y, Michailidis G, Li JZ. Critical limitations of consensus clustering in class discovery. Sci Rep. 2014: 6207 [PMID: 25158761 10.1038/srep06207: 10.1038/srep06207]
10 Wilkerson MD, Hayes DN. ConsensusClusterPlus: a class discovery tool with confidence assessments and item tracking. Bioinformatics. 2010: 1572
11 Pattharanitima P, Thongprayoon C, Petnak T, Srivali N, Gembillo G, Kaewput W, Chesdachai S, Vallabhajosyula S, O'Corragain OA, Mao MA, Garovic VD, Qureshi F, Dillon JJ, Cheungpasitporn W. Machine Learning Consensus Clustering Approach for Patients with Lactic Acidosis in Intensive Care Units. J Pers Med. 2021: [PMID: 34834484 10.3390/jpm11111132: 10.3390/jpm11111132]
12 Thongprayoon C, Dumancas CY, Nissaisorakarn V, Keddis MT, Kattah AG, Pattharanitima P, Petnak T, Vallabhajosyula S, Garovic VD, Mao MA, Dillon JJ, Erickson SB, Cheungpasitporn W. Machine Learning Consensus Clustering Approach for Hospitalized Patients with Phosphate Derangements. J Clin Med. 2021: [PMID: 34640457 10.3390/jcm10194441: 10.3390/jcm10194441]
13 Thongprayoon C, Hansrivijit P, Mao MA, Vaitla PK, Kattah AG, Pattharanitima P, Vallabhajosyula S, Nissaisorakarn V, Petnak T, Keddis MT, Erickson SB, Dillon JJ, Garovic VD, Cheungpasitporn W. Machine Learning Consensus Clustering of Hospitalized Patients with Admission Hyponatremia. Diseases. 2021: [PMID: 34449583 10.3390/diseases9030054: 10.3390/diseases9030054]
14 Thongprayoon C, Jadlowiec CC, Kaewput W, Vaitla P, Mao SA, Mao MA, Leeaphorn N, Qureshi F, Pattharanitima P, Qureshi F, Acharya PC, Nissaisorakarn P, Cooper M, Cheungpasitporn W. Distinct Phenotypes of Kidney Transplant Recipients in the United States with Limited Functional Status as Identified through Machine Learning Consensus Clustering. J Pers Med. 2022: [PMID: 35743647 10.3390/jpm12060859: 10.3390/jpm12060859]
15 Thongprayoon C, Kattah AG, Mao MA, Keddis MT, Pattharanitima P, Vallabhajosyula S, Nissaisorakarn V, Erickson SB, Dillon JJ, Garovic VD, Cheungpasitporn W. Distinct phenotypes of hospitalized patients

with hyperkalemia by machine learning consensus clustering and associated mortality risks. Qjm. 2022: 442 [PMID: 34270780 10.1093/qjmed/hcab194: 10.1093/qjmed/hcab194]
16 Thongprayoon C, Mao MA, Kattah AG, Keddis MT, Pattharanitima P, Erickson SB, Dillon JJ, Garovic VD, Cheungpasitporn W. Subtyping hospitalized patients with hypokalemia by machine learning consensus clustering and associated mortality risks. Clin Kidney J. 2022: 253 [PMID: 35145640 10.1093/ckj/sfab190: 10.1093/ckj/sfab190]
17 Thongprayoon C, Mao MA, Keddis MT, Kattah AG, Chong GY, Pattharanitima P, Nissaisorakarn V, Garg AK, Erickson SB, Dillon JJ, Garovic VD, Cheungpasitporn W. Hypernatremia subgroups among hospitalized patients by machine learning consensus clustering with different patient survival. J Nephrol. 2022: 921 [PMID: 34623631 10.1007/s40620-021-01163-2: 10.1007/s40620-021-01163-2]
18 Thongprayoon C, Mao SA, Jadlowiec CC, Mao MA, Leeaphorn N, Kaewput W, Vaitla P, Pattharanitima P, Tangpanithandee S, Krisanapan P, Qureshi F, Nissaisorakarn P, Cooper M, Cheungpasitporn W. Machine Learning Consensus Clustering of Morbidly Obese Kidney Transplant Recipients in the United States. J Clin Med. 2022: [PMID: 35743357 10.3390/jcm11123288: 10.3390/jcm11123288]
19 Thongprayoon C, Nissaisorakarn V, Pattharanitima P, Mao MA, Kattah AG, Keddis MT, Dumancas CY, Vallabhajosyula S, Petnak T, Erickson SB, Dillon JJ, Garovic VD, Kashani KB, Cheungpasitporn W. Subtyping Hyperchloremia among Hospitalized Patients by Machine Learning Consensus Clustering. Medicina (Kaunas). 2021: [PMID: 34577826 10.3390/medicina57090903: 10.3390/medicina57090903]
20 Thongprayoon C, Sy-Go JPT, Nissaisorakarn V, Dumancas CY, Keddis MT, Kattah AG, Pattharanitima P, Vallabhajosyula S, Mao MA, Qureshi F, Garovic VD, Dillon JJ, Erickson SB, Cheungpasitporn W. Machine Learning Consensus Clustering Approach for Hospitalized Patients with Dysmagnesemia. Diagnostics (Basel). 2021: [PMID: 34829467 10.3390/diagnostics11112119: 10.3390/diagnostics11112119]
21 Thongprayoon C, Vaitla P, Jadlowiec CC, Leeaphorn N, Mao SA, Mao MA, Pattharanitima P, Bruminhent J, Khoury NJ, Garovic VD, Cooper M, Cheungpasitporn W. Use of Machine Learning Consensus Clustering to Identify Distinct Subtypes of Black Kidney Transplant Recipients and Associated Outcomes. JAMA Surg. 2022: e221286 [PMID: 35507356 10.1001/jamasurg.2022.1286: 10.1001/jamasurg.2022.1286]
22 Thongprayoon C, Vaitla P, Nissaisorakarn V, Mao MA, Genovez JLZ, Kattah AG, Pattharanitima P, Vallabhajosyula S, Keddis MT, Qureshi F, Dillon JJ, Garovic VD, Kashani KB, Cheungpasitporn W. Clinically Distinct Subtypes of Acute Kidney Injury on Hospital Admission Identified by Machine Learning Consensus Clustering. Med Sci (Basel). 2021: [PMID: 34698185 10.3390/medsci9040060: 10.3390/medsci9040060]
